# How can implementation intentions be used to modify gambling behavior?

**DOI:** 10.3389/fpsyg.2022.957120

**Published:** 2022-11-09

**Authors:** Tom St Quinton

**Affiliations:** School of Psychology and Therapeutic Studies, Faculty of Social and Health Sciences, Leeds Trinity University, Leeds, United Kingdom

**Keywords:** implementation intentions, motivation, gambling, planning, intention

## Abstract

Problem gambling can cause significant harm, yet rates of gambling continue to increase. Many individuals have the motivation to stop gambling but are unable to transfer these positive intentions into successful behavior change. Implementation intentions, which are goal-directed plans linking cues to behavioral responses, can help bridge the gap between intention and many health behaviors. However, despite the strategy demonstrating popularity in the field of health psychology, its use in the area of gambling research has been limited. This mini review illustrates how implementation intentions can be used to facilitate change in gambling behavior. Adopting the strategy could help reduce the number of people with gambling problems.

## Introduction

Gambling disorders, problem gambling, and excessive gambling can be detrimental to a person’s health. It can lead to negative consequences such as depression, unemployment, financial distress, relationship problems, and criminal behavior (Afifi et al., 2016; Bond et al., 2016; Adolphe et al., 2019; Muggleton et al., 2021). Problem gambling can co-occur with other behavioral addictions (Kessler et al., 2008; Konkolÿ Thege et al., 2016), have considerable impact on close ones (Goodwin et al., 2017), and potentially lead to suicide ideation and behavior (Wardle and McManus, 2021). It is estimated that problem gamblers account for between 0.1 and 5.8% of the population (Williams et al., 2012; Calado and Griffiths, 2016).

Problem gambling has been an issue in Western society for decades, though the availability and opportunity to gamble has increased significantly over the past few years (Abbott, 2020). For example, the recent growth in internet and online gambling has had a major impact on the gambling experience (Gainsbury, 2015). Among the many advantages, internet gambling is highly accessible, convenient to users, and can be undertaken anonymously (Gainsbury et al., 2012). There has also been a rapid rise in gambling advertisements with gambling companies offering free bets, enhanced odds, sign up bonuses, and money back guarantees (Clemens et al., 2017; Rawat et al., 2020). Due to the availability of gambling and the influence of promotional campaigns, it is predicted that gambling is not going to abate in the future (Hodgins and Stevens, 2021). Thus, there is a need for interventions to successfully intervene on and reduce gambling behavior.

### Motivation toward gambling

Understanding and explaining human behavior can be achieved using social cognition theories and models, such as the theory of planned behavior (Ajzen, 1991), the theory of reasoned action (Fishbein and Ajzen, 1975), and social cognitive theory (Bandura, 1986). These approaches identify the mechanisms through which psychological beliefs and determinants influence behavior. As well as understanding behavior, these theories can also inform the development of behavior change interventions. Specifically, the theories can identify the important and modifiable psychological determinants that interventions should target. For illustrative purposes and due to its popularity in the health domain, the theory of planned behavior will be the focus of this paper.

The theory of planned behavior places intention as the proximal determinant of behavior. Intention represents a person’s motivation and willingness to engage in the behavior and is determined by attitude (i.e., behavioral evaluations), subjective norm (i.e., perceived approval of others), and perceived behavioral control (i.e., perceived control over the behavior). A large body of research has attested to the utility of the theory in understanding why people do or do not engage in health promoting or health risk behaviors. The theory has been found to account for between 40 and 45% of the variance in intention and 19–36% of the variance in behavior (e.g., Armitage and Conner, 2001; Hagger et al., 2002; McEachan et al., 2011). Although research applied to gambling has been limited, some studies have used the theory to understand gambling intentions and behavior (e.g., Martin et al., 2010; Wu and Tang, 2012; St-Pierre et al., 2015; St Quinton, 2021). Wu and Tang (2012) and Martin et al. (2010) found gambling intentions to be predicted by attitude, subjective norm, and perceived behavioral control. Similarly, St-Pierre et al. (2015) identified intention to be predicted by attitude and perceived behavioral control. In relation to gambling behavior, research has found intention to be a significant predictor (Wu and Tang, 2012; St Quinton, 2021).

### The intention-behavior gap

The theory of planned behavior has accounted for impressive variance in intention towards health behaviors, including gambling. However, there is a well-known gap between what people intend to do and what actually occurs (Sheeran and Webb, 2016). Experimental evidence has demonstrated that medium-to-large changes in intention results in only small-to-medium changes in behavior (Webb and Sheeran, 2006). The successful enaction of intention often involves overcoming setbacks, not giving in to temptations, and staying on track (Heckhausen and Gollwitzer, 1987; Schwarzer and Luszczynska, 2008). In relation to gambling, individuals may have the intention to limit or abstain from the behavior but nevertheless proceed to gamble. Indeed, evidence has shown that a large proportion of those with the motivation or intention to stop gambling often relapse (Hodgins and el-Guebaly, 2004; Smith et al., 2015), and a significant number drop out from gambling-related treatment (Pfund et al., 2021). Given the theory of planned behavior specifies intention as the proximal determinant of behavior, the model has been suggested to be unable to account fully for behavior (Sniehotta et al., 2014). The question remains as to how those intending to refrain or abstain from gambling can be facilitated in doing so.

## Implementation intentions

To account for the gap between intention and behavior, various strategies and models have been developed. In health psychology, research has found the benefits of planning, specifically implementation intentions. Implementation intentions are goal-directed plans formed based on *what*, *when*, *where*, and *how* the behavior will be undertaken. To develop an implementation intention, two important aspects must be considered (Gollwitzer, 2014). First, one must identify a cue or critical situation. These can be cues facilitating the behavior or cues related to barriers to be overcome. Second, an appropriate behavioral response must be linked to the situation. These behavioral responses are behaviors to be undertaken once the cue is encountered. Implementation intentions are effective because deliberately identifying cues improves perceptual readiness and once encountered, automatically elicits the behavioral response (Webb and Sheeran, 2004; Gollwitzer, 2014). The effectiveness of these plans can be increased when an “if/then” format is used to link the cue to the response. For example, a person wishing to stop gambling may state “If I am asked to go to a casino, then I will remind myself of the money I will likely lose.” Here, encountering the proposition to attend the casino will automatically elicit a reminder of the potential negative consequences.

It is important to note that an implementation intention is not a motivational strategy, and its effectiveness is not due to increased motivation (Webb and Sheeran, 2008). That is because planning focuses on increasing opportunities to undertake the behavior, not on increasing motivation toward the behavior. An individual without a positive intention is unlikely to strengthen their motivation due to developing a plan. However, the effectiveness of planning depends on the presence of motivation. That is, the extent to which planning is successful depends on a person’s willingness to either engage or not engage in the focal behavior. In fact, the effectiveness of implementation intentions can be improved in the presence of strong intentions (Prestwich and Kellar, 2014). Thus, a pre-requisite to planning is a (strong) behavioral intention or (strong) motivation toward the behavior.

Implementation intentions can be self-generated where the researcher or health provider instructs the person to develop their own plans. Specifically, they are asked to identify situational cues and link with appropriate behavioral responses. Given the person develops their own cues and responses, commitment to the plans can be enhanced (Sniehotta, 2009). Alternatively, implementation intentions can be provided by the researcher or health provider. Armitage (2008) developed volitional help sheets which include a number of situational cues and behavioral responses. The individual is instructed to make links between the cues and outcomes deemed most relevant or salient to them. Although this does not guarantee the cues and responses will be applicable to all, this does circumvent any difficulty the person may have in generating their own plans.

Evidence has demonstrated implementation intentions to be effective in promoting behavior (Gollwitzer and Sheeran, 2006), including those related to health (Prestwich et al., 2003; Gollwitzer and Sheeran, 2006; Hagger and Luszcynska, 2014; Presseau et al., 2017). Gollwitzer and Sheeran (2006) showed implementation intentions had moderate-to large effects on health behaviors. Presseau et al. (2017) identified planning had a small-to-medium effect on objectively measured health behavior and a medium effect on self-reported health behavior. Despite these considerable behavioral effects, the use of implementation intentions has been limited in gambling research (Rodda et al., 2020). For example, a systematic review conducted by St Quinton et al. (2022) identified 18 strategies were used in interventions targeting adolescent gambling. However, planning was not included in any intervention. Furthermore, Rodda et al. (2018) did find action planning was used by problem gamblers during online forums. However, it is unclear whether the plans were developed and enacted in the form of implementation intentions. Finally, Rodda et al. (2017) found that despite the uptake of many behavioral strategies, including planning, their use in gambling prevention is often not maintained. Therefore, there is a gap for implementation intentions to be used in this area.

It is worth noting that implementation intentions share similarities with other treatments used to modify risky behavior, such as gambling. For example, therapeutic approaches such as cognitive-behavioral therapy and motivational interviewing often attempt relapse prevention by identifying triggers. However, there are important differences between these approaches and implementation intentions. For example, although clients undertaking cognitive behavioral therapy are often asked to specify when and where they will strive for their goals, therapists rarely prompt clients to link a critical cue with a goal-directed response (Duhne et al., 2020). Moreover, unlike these treatment approaches, the adoption of implementation intentions does not necessitate communication between a client and interventionist (Wittleder et al., 2019). Evidence has also shown implementation intentions can be more effective than these therapeutic approaches (e.g., Varley et al., 2011; Mutter et al., 2020).

### Implementation intentions and gambling

Individuals intending to quit or limit their gambling often fail to follow through on these good intentions (Hodgins, and el-Guebaly, N., 2004; Smith et al., 2015). There are many factors that can interfere with these intentions. However, adopting implementation intentions could prevent motivation to refrain from gambling being derailed. Examples of how implementation intentions could be used are described next, but additional situations and responses are presented in Table 1.

**Table 1:** Examples of situations, responses, and implementation intentions related to gambling prevention.

Situations/cues	Responses	Implementation intention
Friends	Polite refusal	If my friends ask me to go to the casino, then I will politely decline and inform them of my motivation to stop gambling
Work colleague	Recall success	If I am asked to go to the bookmakers by a colleague, then I will recall how long I have abstained from gambling
Sports team	Importance of saving	If my sports team attend a bookmaker before a match, then I will remember the money I need to save
Anger	Walking	If I am angry and have the urge to gamble, then I will go for a walk instead
Sad	Speaking with a friend	If I am sad and contemplate gambling, then I will phone my friend and tell them how I feel
Stressed	Recall relationship issues	If I am angry and consider having a bet, I will remember the damage gambling causes to my relationship
Boredom	Watch television	If I am bored and am tempted to gamble, then I will watch my favorite TV program
Happy	Mood reminder	If I am happy and contemplate gambling, then I will remind myself that gambling will negatively influence my mood
Feeling lucky	Reminder of odds	If I am feeling lucky and contemplate gambling, then I will remind myself that the odds are not in my favor and I am likely to lose
Specific days	Lose control	If I have the urge to gamble after work on Fridays, then I will recall how quickly I can spiral and lose money
Specific times	Holiday reminder	If I have the desire to bet during the evening, then I will remind myself of the importance of saving money for my upcoming family holiday
Location	Alternative route	If I see a betting shop on my way home, then I will find an alternative route
Losing money	Bet limit	If I lose £10 on the slot machines, then I will stop gambling immediately
Gambling advertisement	Vacate area	If a betting advert comes on the television, then I will leave the room
Pop-ups	Action to close	If I encounter a gambling pop up, then I will immediately close it
Account log in	Limit deposits	If I log onto my betting account, then I will deposit no more than £5
Mobile phone	Download prevention	If I am on my phone, I will not download any gambling apps
Casino	Friend support	When I am in a casino, I will ensure that I ask my friend to stop me from gambling
Bookmakers	Avoidance	When I am in the bookmakers, I will stand on the opposite side of the room to the slot machines
Horse racing	Recall mood	When I am at horse racing, I will remember how bad gambling makes me feel

Gambling is often undertaken as a social activity and can be influenced by the behaviors and actions of others (Zhai et al., 2017; Mazar et al., 2018). Such referents can include friends, work colleagues, and social groups (i.e., sports teams, book clubs, and gym classes). Although these referents can support gambling abstinence, they may also have a negative influence by encouraging it. However, the negative influence of these groups can be subsided through implementation intentions. Consider a person invited to a casino by a group of friends. Now, this person could develop a plan that responds to the invitation. For example, the individual may state “If my friends ask me to go to the casino, then I will politely decline and inform them of my motivation to stop gambling.” In this way, encountering the situation (the invitation) would automatically lead to a positive behavioral response (the polite refusal). The implementation intention therefore protects the motivation or intention to abstain from gambling without having the need for conscious deliberation. As another example, a colleague may frequently visit a bookmaker during lunchtime. When asked by the colleague whether they would like to accompany them one day, the implementation intention “If I am asked to go to the bookmakers by a colleague, then I will recall how much money I have saved through gambling abstinence” could facilitate a positive behavioral response. Again, the plan put in place means the response is guided automatically when the situation is encountered.

These examples use implementation intentions to avoid entering a gambling facility. However, this may not always be preferred or needed. For example, in example 1, the individual may believe that refusing to go along to the casino with friends is likely to cause upset or disappointment. However, an implementation intention could be developed to elicit positive behavioral responses in these locations (e.g., “When I am in a casino, I will ensure that I ask my friend to stop me from gambling”). Similarly, the person in example 2 may feel confident going into the bookmakers but have concerns about abstaining from the slot machines. To facilitate abstinence, a plan such as “When I am in the bookmakers, I will stand on the opposite side of the room to the slot machines” could be used. Additionally, these examples relate to intentions to abstain from gambling, but the same logic would apply to limiting gambling. For example, example one could be modified to “If I am asked to go to the bookmakers by a colleague, then I will accept but put no more than £5 into the machine” or “If I lose £5 on the slot machine, then I will stop gambling immediately.”

In addition to social influences, gambling can be triggered by specific negative emotions, such as stress, boredom, or anger (Buchanan et al., 2020; Kılıç et al., 2020). Implementation intentions can be used overcome these emotions. An individual prone to gambling when angry could plan “If I am angry and have the urge to gamble, then I will go for a walk instead.” In addition to negative emotions, gambling can be influenced by positive emotions (Rogier et al., 2022). A plan could be developed to prevent such feelings leading to gambling. For example, an implementation intention could state “If I am happy and contemplate gambling, then I will remind myself that gambling will negatively influence my mood.”

There may be specific times or days when gambling are more likely to occur (Morasco et al., 2007). For example, an individual could be more susceptible to gambling during the evening or after finishing work on a particular day. Implementation intentions such as “If I have the urge to gamble after work on Fridays, then I will recall how quickly I can spiral and lose money” and “If I have the desire to bet during the evening, then I will remind myself of the importance of saving money for my upcoming family holiday” could protect motivation toward abstinence. Finally, planning could be used to limit the influence of gambling advertisements, promotions, and pop-ups. Consider a person sitting watching a football match on television and a gambling advert appears on the screen with various enhanced odds for the match. This could make the person want to have a bet on the match. However, the implementation intention “If a betting advert comes on the television, then I will leave the room” could deter them from doing so.

These examples and those provided in Table 1 are not exhaustive as there will be many other circumstances and situations associated with gambling. Additionally, variability will exist in the effect of eliciting cues. For example, one individual may be inclined to gamble when stressed whereas another person may not. Moreover, the automatic behavioral responses will not be pertinent for each person; two people may be tempted to gamble in the evening, but only one may be influenced by a reminder that they are saving for a family holiday. It is therefore important that plans are relevant to the person wishing to abstain from gambling. Specifically, the situation and cues need to be considered carefully to elicit positive behavioral change. Plan effectiveness is also likely to be determined by the severity of the gambling problem. For example, planning for what happens when entering the casino may not be effective those with a severe gambling problem.

### Implications for gambling

Abstaining from or limiting gambling can be a difficult challenge, even in the presence of strong motivation. Planning how this is going to be achieved can produce effective behavior change. Several recommendations can be made for the use of implementation intentions.

It is important that people are motivated to either limit or refrain from future gambling. That is because without an intention to do so, planning is unlikely to be successful. In the absence of a positive intention, motivational models, such as the theory of planned behavior, should be sought. These models can identify why a person is lacking motivation to change, and interventions can be developed accordingly. For example, some individuals may perceive odds to be in their favor or believe roulette outcomes can be predicted. In these instances, education could be useful to modify these erroneous attitudes.

In the absence of an intention to change, focus should be given to motivation. But the ceiling of motivation means this is usually insufficient to modify gambling behavior. Interventions should therefore introduce planning strategies to facilitate positive intentions. Given the ease of which planning interventions or instructions can be delivered, there is vast potential for its use in the gambling domain. Research in health psychology has recently adopted the use of technology to deliver behavioral interventions. For example, popularity has risen in interventions adopting a mobile device (Steinhubl et al., 2015). Given the number of people using mobile phones to gamble (James et al., 2017); this could be an effective mode to prevent it. This may be especially useful given a large proportion of gamblers are unwilling to seek face-to-face help (Gainsbury et al., 2014). Communication could be made through text messages, emails, or mobile applications. For example, a text message could notify the user to develop an implementation intention, or a mobile application could instruct on how gambling abstinence can be achieved through planning. Bi-directional communication could be adopted whereby the user responds to such requests, and the implementation intentions are checked by the provider.

The optimal effectiveness of implementation intentions depends on ensuring aspects of fidelity. Firstly, it is important that the planning instructions are delivered in accordance with the strategy. If recommending implementation intentions, clear instructions need to be provided related to the identification of situations and behavioral responses. If, however, cues and behavioral responses are provided, then careful consideration must be given to ensure these are prominently associated with gambling. Secondly, it is important that the person wishing to abstain from gambling adheres to the instructions given. For example, failure to pair risky situations with critical responses would undermine the psychology underlying the effectiveness of planning.

## Conclusion

Abstaining from or limiting gambling behavior is difficult and motivation is often insufficient. A useful strategy facilitating motivation is implementation intentions, a specific type of planning. The importance of both motivation and implementation intentions can be illustrated by considering the following: there is one individual with strong motivation to stop gambling. Now, this individual does not plan for circumstances that may deter influence motivation to refrain from gambling. When encountering many unanticipated events, the individual fails to implement the intention by proceeding to gamble. There is a second individual with the same strong intention to refrain from gambling. Unlike the first individual, this person has also developed specific implementation intentions after considering potential barriers. When this person encounters the same unanticipated events as example 1, they are able overcome them and follow through on their intention to abstain from gambling. Therefore, those with a strong intention to refrain from gambling plus implementation intentions may be better placed to reduce or abstain from gambling. Consequently, implementation intentions should be utilized by those wishing to modify their gambling behavior.

## Author contributions

The author confirms being the sole contributor of this work and has approved it for publication.

## Conflict of interest

The author declares that the research was conducted in the absence of any commercial or financial relationships that could be construed as a potential conflict of interest.

## Publisher’s note

All claims expressed in this article are solely those of the authors and do not necessarily represent those of their affiliated organizations, or those of the publisher, the editors and the reviewers. Any product that may be evaluated in this article, or claim that may be made by its manufacturer, is not guaranteed or endorsed by the publisher.
